# Proteolysis-a characteristic of tumor-initiating cells in murine metastatic breast cancer

**DOI:** 10.18632/oncotarget.11309

**Published:** 2016-08-16

**Authors:** Larissa E. Hillebrand, Fee Bengsch, Jochen Hochrein, Jan Hülsdünker, Julia Bender, Marie Follo, Hauke Busch, Melanie Boerries, Thomas Reinheckel

**Affiliations:** ^1^ Institute of Molecular Medicine and Cell Research, Medical Faculty, Albert-Ludwigs-University Freiburg, Freiburg, Germany; ^2^ Faculty of Biology, Albert-Ludwigs-University Freiburg, Freiburg, Germany; ^3^ BIOSS Centre for Biological Signalling Studies, Freiburg, Germany; ^4^ Systems Biology of the Cellular Microenvironment Group, Institute of Molecular Medicine and Cell Research, Albert-Ludwigs-University Freiburg, Freiburg, Germany; ^5^ Comprehensive Cancer Center Freiburg, Freiburg, Germany; ^6^ Department of Hematology/Oncology, Core Facility, University Medical Center, Freiburg, Germany; ^7^ German Cancer Consortium (DKTK), German Cancer Research Center (DKFZ), Heidelberg, Germany

**Keywords:** breast cancer, degradome, matrix metalloproteinase, proteolysis

## Abstract

Tumor initiating cells (TICs) have been identified and functionally characterized in hematological malignancies as well as in solid tumors such as breast cancer. In addition to their high tumor-initiating potential, TICs are founder cells for metastasis formation and are involved in chemotherapy resistance. In this study we explored molecular pathways which enable this tumor initiating potential for a cancer cell subset of the transgenic MMTV-PyMT mouse model for metastasizing breast cancer. The cell population, characterized by the marker profile CD24^+^CD90^+^CD45^−^, showed a high tumorigenicity compared to non-CD24^+^CD90^+^CD45^−^ cancer cells in colony formation assays, as well as upon orthotopic transplantation into the mammary fat pad of mice. In addition, these orthotopically grown CD24^+^CD90^+^CD45^−^ TICs metastasized to the lungs. The transcriptome of TICs freshly isolated from primary tumors by cell sorting was compared with that of sorted non-CD24^+^CD90^+^CD45^−^ cancer cells by RNA-seq. In addition to more established TIC signatures, such as epithelial-to-mesenchymal transition or mitogen signaling, an upregulated gene set comprising several classes of proteolytic enzymes was uncovered in the TICs. Accordingly, TICs showed high intra- and extracellular proteolytic activity. Application of a broad range of protease inhibitors to TICs in a colony formation assay reduced anchorage independent growth and had an impact on colony morphology in 3D cell culture assays. We conclude that CD24^+^CD90^+^CD45^−^ cells of the MMTV- PyMT mouse model possess an upregulated proteolytic signature which could very well represent a functional hallmark of metastatic TICs from mammary carcinomas.

## INTRODUCTION

Malignant tumors consist of heterogeneous cancer cell populations [1]. Understanding the functional and therapeutic implications of this heterogeneity has become a major challenge for basic and translational cancer research [2, 3]. The cancer stem cell hypothesis is increasingly recognized as an explanation for the intratumoral heterogeneity of cancer cells [4]. Cancer stem cells were first identified in hematological malignancies by Bonnet and Dick [5]. Nonetheless, this concept has also been established for a number of epithelial derived cancers, such as colon, prostate, breast, and pancreatic carcinomas [3, 6–9]. Essentially, cancer stem cells have the ability to asymmetrically divide, which results in self-renewal of the tumorigenic stem cell population and a large mass of cancer cells that have no, or only a limited, ability to establish new tumors [10, 11]. In addition, malignant stem cells from carcinomas are thought to be motile and, therefore, able to establish metastases in distant organs [12]. Due to their cancer-forming ability, and in order to distinguish them from physiological stem cells in tissues, these cells are often referred to as tumor initiating cells (TICs); a term which we will use hereafter.

Based on a variety of specific cell surface markers, TICs can be isolated from solid tumors via fluorescence- activated cell sorting (FACS) and subsequently functionally characterized *in vitro* and *in vivo* [13–15]. The first TICs in human breast cancers were identified based on the cell surface makers CD44^+^CD24^-/low^ [13]. Different cell surface markers have been used to identify TICs in specific murine breast cancer models, including CD29, CD61, Epcam and CD49f [13–16]. In the MMTV-Wnt1 model for breast cancer TICs can be isolated based on the cell surface markers CD24^+^ and CD90^+^ (Thy1) and the exclusion of CD45 positive leukocytes [15]. These cells showed high tumorigenicity upon injection of only 50 cells into the mammary fat pad of female mice. Using these markers, TICs have also been obtained from the MMTV-PyMT mouse model of metastatic breast cancer, which were highly efficient in forming colonies in the lungs upon tail vein injection [17]. More recently, MMTV-PyMT derived CD24^+^CD90^+^ cells have been instrumental to demonstrate the metastasis-supporting function of neutrophil granulocytes [18] and for the elucidation of interaction of stroma and cancer cells during metastatic colonization [19]. However, the tumorigenic potential of the MMTV-PyMT derived CD24^+^CD90^+^ cell population by limiting dilution assays *in vivo* has not been reported.

In this study, CD24^+^CD90^+^CD45^−^ cells from primary MMTV-PyMT breast tumors were isolated and their clonogenic and tumorigenic abilities were characterized in detail. We found evidence for a potent TIC population. Moreover, RNA-seq analysis of freshly sorted TICs compared to less tumorigenic cancer cells revealed a difference in molecular profiles. Notably, a strong signature of increased expression of various protease genes in TICs was identified. As proteolysis is known to promote growth and invasion in cancer [1, 20, 21], we set out to demonstrate the proteolytic capacity of MMTV-PyMT derived TICs. Protease inhibitors reduced anchorage independent growth as well as collagen cleavage of TICs. Our findings give insight into the proteolytic network of TICs and suggest proteolysis as a novel characteristic of tumor- initiating breast cancer cells.

## RESULTS

### CD24^+^CD90^+^ cells isolated from MMTV-PyMT mice display high tumorigenic potential

**Figure 1:**
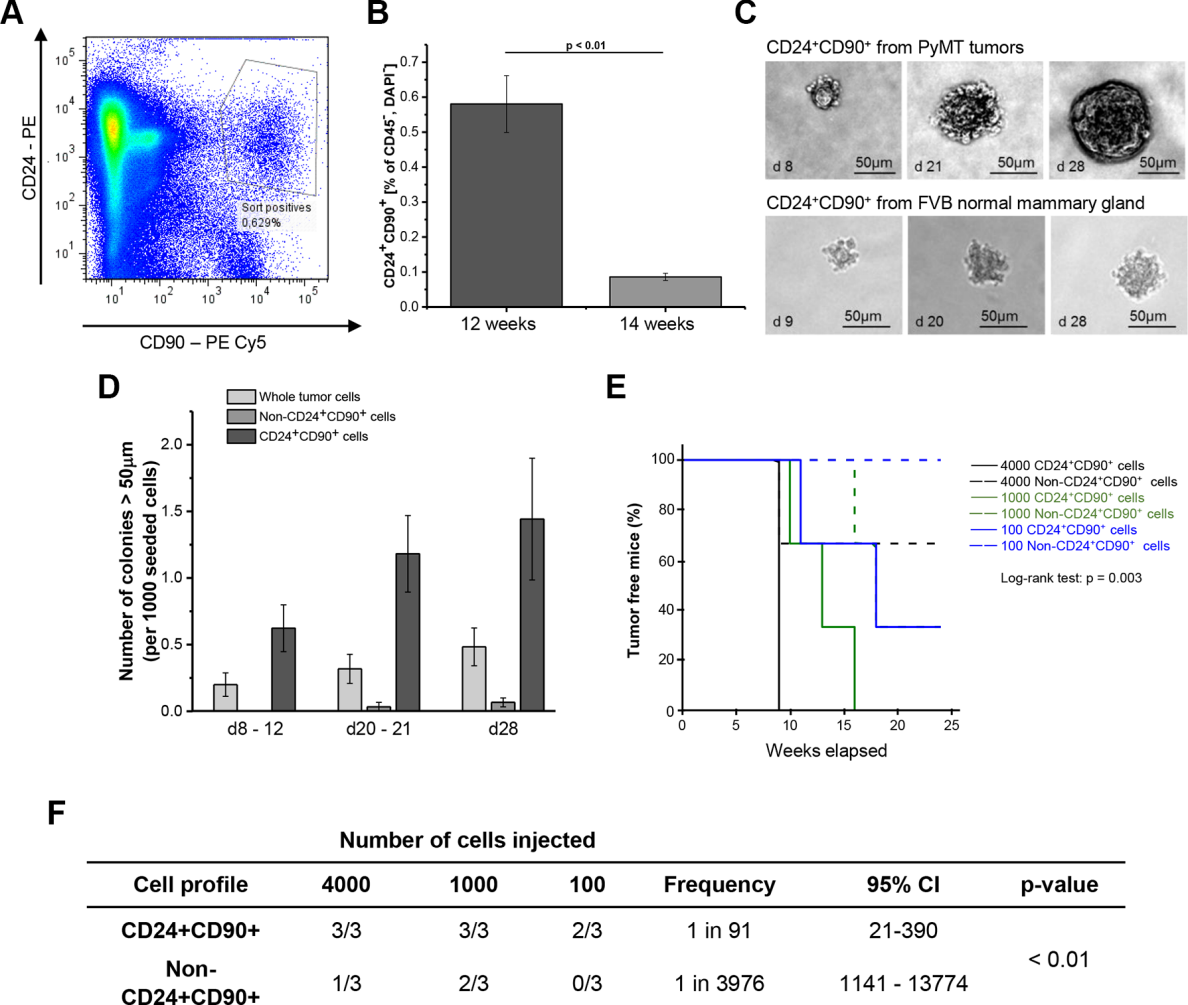
Tumorigenic properties of CD24^+^CD90^+^ cells (**A**) Fluorescence- activated cell sorting (FACS) plot of whole tumor single cell suspension from a representative primary MMTV-PyMT tumor. Cells are stained for CD24 and CD90 and depleted for CD45 and 4′,6-Diamidin-2-phenylindol; (**B**) Frequency of CD24^+^CD90^+^ cells isolated from 12 week (*n* = 21) and 14 week (*n* = 5) old tumor mice. (**C**) Colonies grown from CD24^+^CD90^+^ sorted cells from MMTV-PyMT tumors and FVB normal mammary gland; (**D**) Colony forming capacity of CD24^+^CD90^+^ cells (*n* = 3) compared to whole tumor cell suspension (*n* = 3) and non-CD24^+^CD90^+^ cell population (*n* = 3); (**E**) Kaplan- Meier plot of incidence of TIC- derived tumors after injection of 4000 (black), 1000 (olive) or 100 (blue) CD24^+^CD90^+^ cells (straight lines) or non-CD24^+^CD90^+^ tumor cells (dashed lines); (**F**) CD24^+^CD90^+^ cells and non-CD24^+^CD90^+^ tumor cells from 3 different tumors were injected in cell dosages listed. Three injections were performed (denominator). Number of resultant tumors can be seen in the numerator of the table. Frequency = tumorigenic frequency, 95% CI = 95% confidence interval.

Tumor cells positive for the cell surface markers CD24 and CD90 are known for their high tumorigenicity in the transgenic MMTV-Wnt1 mouse model and have been called cancer stem cells [15]. Here, CD24^+^CD90^+^ cancer cells from primary breast tumors of MMTV-PyMT mice were obtained by FACS. To avoid leukocyte contamination, cells expressing the common leukocyte antigen CD45 were always excluded from the CD24^+^CD90^+^ population, which resulted in a double-positive population constituting 0.11 to 1.4 percent of the CD45 negative cells in the tumor (Figure 1A).

Tumor growth in the MMTV-PyMT mouse model is induced by puberty [22]. Subsequently, the breast tissue undergoes a series of consecutive transformation events from initially benign lesions to invasive carcinomas. In the FVB/N mouse background individual tumors reach a size of about 1 cm^3^ and most of the mice develop lung metastases by 14 weeks of age [22]. Therefore, this time point was set as the final stage for analysis. However, the percentage of CD24^+^CD90^+^ cells was very low (0.086%) in large tumors at 14 weeks. In contrast, the amount of double-positive tumor cells was almost seven times higher (0.58%) in tumors from 12-week-old mice (Figure 1B). This difference of the relative pool of CD24^+^CD90^+^ cells in 12 and 14 week MMTV-PyMT mice is likely due to the exponential tumor growth occurring in this cancer model between the age of 11 and 14 weeks. Apparently the CD24^+^CD90^+^ cells are not expanding with the same speed as the other cell populations in the cancer. Consequently, all further experiments were conducted with CD24^+^CD90^+^ cells from 12-week-old mice.

A typical feature of tumor initiating cells is anchorage- independent growth and colony formation *in vitro* [23, 24]. Therefore, CD24^+^CD90^+^ cells from MMTV-PyMT breast cancers, as well as CD24^+^CD90^+^ cells from FVB normal mammary gland, were grown in a soft agar matrix. Both cell types formed colonies after nine days, which is a typical characteristic for stem cell activity [25]. Colonies derived from PyMT tumors were well-defined, while colonies derived from normal mammary gland were smaller and more loosely connected aggregates of cells (Figure 1C). After 28 days of culture the breast cancer derived colonies were highly compact and twice the size of the normal mammary gland derived colonies. Subsequently, colony formation of CD24^+^CD90^+^ tumor cells was compared with both a sorted non-CD24^+^CD90^+^ tumor cell population that was subject to live/death sorting as well as depleted of CD45^+^ cells, and a whole tumor cell preparation that was subject to live/death sorting. The colony forming efficiency of the CD24^+^CD90^+^ tumor cells was 3 fold higher compared to whole tumor cells and 24 times higher compared to non-CD24^+^CD90^+^ cells (Figure 1D). Thus, CD24^+^CD90^+^ cells have a comparatively high ability to grow anchorage- independently. This indicates stemness and potential tumorigenicity for CD24^+^CD90^+^ cells derived from MMTV-PyMT breast cancers. From this point on we will denote this cell population as tumor initiating cells (TICs).

The functional gold standard for a TIC is the ability to form tumors *in vivo*. Therefore, 100, 1000 or 4000 TICs, as well as corresponding numbers of non-CD24^+^CD90^+^ tumor cells, were transplanted into the fat pad of either the right or the left fourth mammary gland of immunodeficient Rag2^−/−^ γc^−/−^female mice. After nine weeks, a cell dosage of 4000 TICs produced palpable tumors in three out of three mice, which reached a size of 1 cm^3^ after 13 weeks (Figure 1E and 1F). The tumors were firm and macroscopically similar. One out of three mice developed a cystic tumor from 4000 non-CD24^+^CD90^+^ tumor cells. All of the mice which had received 1000 TICs developed tumors between 10 and 13 weeks after cell transplantation, which reached a final size of 1 cm^3^ approximately 3 weeks later (Figure 1E and 1F). Two mice developed tumors resulting from 1000 non-CD24^+^CD90^+^ tumor cells. These tumors grew slowly and were smaller than 0.5 cm^3^ upon sacrificing the mice 3 weeks after tumor onset. Most importantly, two out of three mice developed tumors upon the injection of only 100 TICs into the mammary fat pad after 11 and 18 weeks (Figure 1E and 1F). In contrast, no tumor development was observed upon the injection of 100 non-CD24^+^CD90^+^ tumor cells. This translates to a tumorigenic frequency of 1 in 91 cells for TICs, which is about 43 × higher than that of non-CD24^+^CD90^+^ cells (Figure 1F). Thus, MMTV-PyMT breast cancer cells with the marker profile CD24^+^CD90^+^ show high tumorigenicity both *in vitro* and *in vivo*, while cells single- or double-negative for these markers do not.

### Tumors derived from CD24^+^CD90^+^ TICs partially resemble the primary tumor and are metastatic

**Figure 2:**
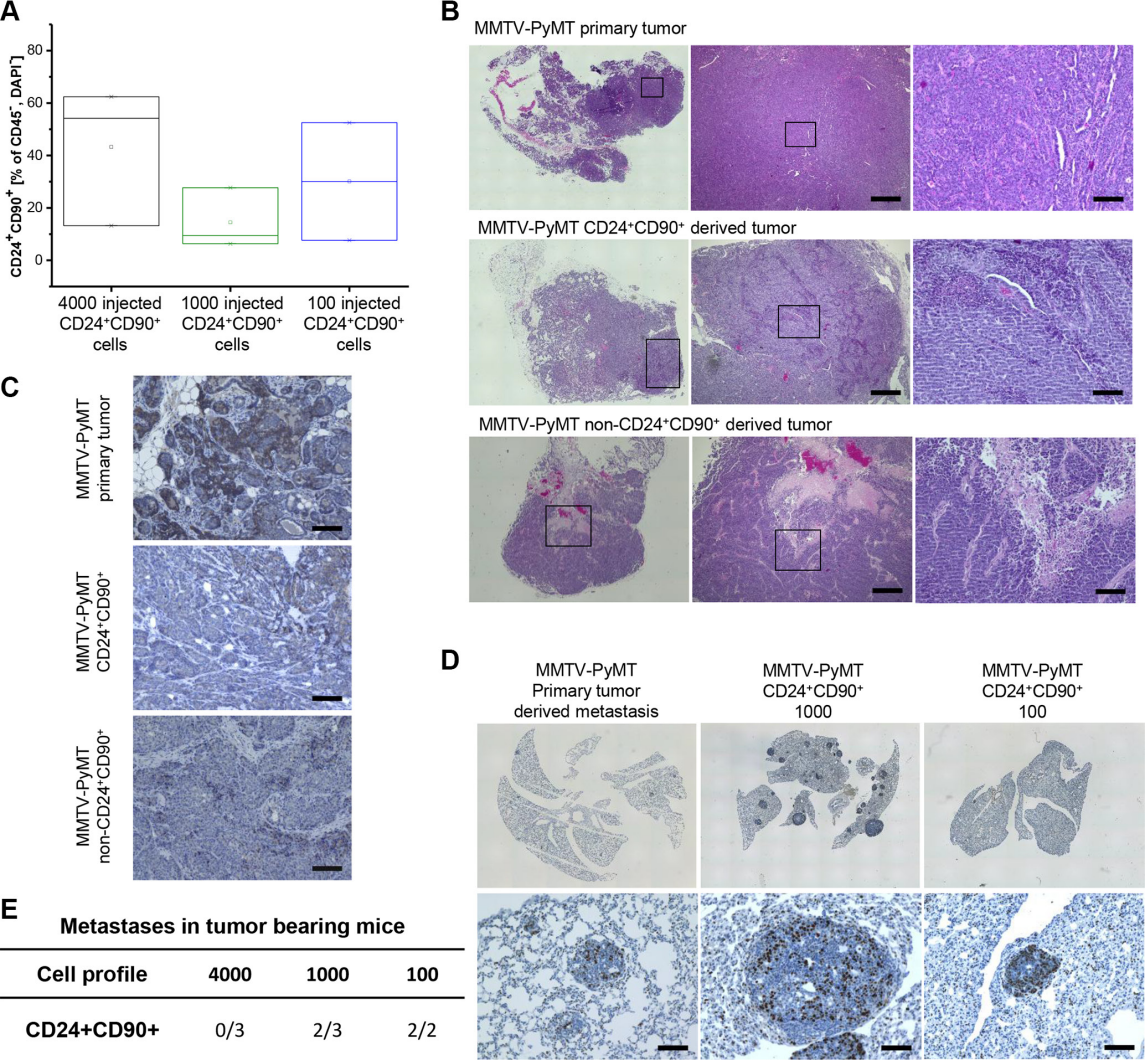
Comparison of primary MMTV-PyMT and TIC-derived tumors and metastasis (**A**) Percentage of CD24^+^CD90^+^ population in secondary TIC-derived tumors (percentage of CD45-, DAPI- population); 4000 injected cells: black; 1000 injected cells: olive, 100 injected cells: blue); (**B**) Hematoxylin eosine (HE) staining of primary MMTV-PyMT and secondary tumors derived from TICs and non-CD24^+^CD90^+^ cells; (**C**) Staining of parental MMTV-PyMT and secondary tumors derived from 100 TICs and 1000 non-CD24^+^CD90^+^ tumor cells for the epithelial marker E-cadherin; (**D**) Ki67 staining of primary MMTV-PyMT tumor-derived and TIC-derived tumor (1000 and 100 cells) metastasis; (**E**) Number of tumor bearing mice with metastasis development in the lungs. Number of occurring tumors can be seen in the denominator, number of mice with resultant metastases can be seen in the numerator of the table. Scale bar equals 100 μm.

Analytical flow cytometry and histological analysis were performed to investigate whether TIC-derived tumors recapitulate the original primary tumor of the MMTV-PyMT model. Flow cytometry profiles revealed a great expansion of TICs in transplanted tumors compared to the TIC population of primary MMTV-PyMT tumors (Figures 2A and 1B). Tumors derived from 100 TICs and 1000 non-CD24^+^CD90^+^ cells partly reflected the histology of the primary tumors, displaying many undifferentiated regions characteristic for MMTV-PyMT tumors (Figure 2B). The transplanted tumors, however, had a less epithelial character than the primary tumors. This is demonstrated with the epithelial adhesion molecule E-cadherin, which was strongly reduced in the TIC-derived tumor specimens as well as non-CD24^+^CD90^+^ derived tumors (Figure 2C). Orthotopic transplantation of 1000 and 100 TICs was followed by spontaneous metastasis formation in the lungs. Thus, tumors derived from TICs are able to disseminate from the primary tumor and colonize distant organs during the time of tumor growth to 1 cm^3^. This might involve early metastatic seeding and a parallel progression of primary tumors and metastases [26]. In their morphology and proliferation state (Ki67 staining) these metastases were comparable to metastases formed in female MMTV-PyMT mice at 14 weeks of age (Figure 2D). Mice with tumors derived from 4000 TICs did not develop metastases. This can be attributed to sacrificing the mice earlier after orthotopic transplantation compared to mice that received less cells and needed more time for tumors to reach the stop criterion of 1 cm^3^. Two out of three mice displayed metastases formation that received 1000 TICs, as well as two out of two mice that received 100 TICs (Figure 2E). Note that 1000 transplanted TICs resulted in a higher metastatic burden than orthotopic transplantation of 100 TICs, although each of the mice was analyzed at a tumor volume of 1 cm^3^.

Taken together, these observations reveal that the TICs under investigation are able to form tumors, which are of an undifferentiated basal-like appearance, and are able to metastasize to the lungs. Thus, CD24^+^CD90^+^ tumor cells are not only tumorigenic, but also metastatic and represent a metastatic TIC population in the MMTV-PyMT model, which is in line with other work on CD24^+^CD90^+^ TICs [17–19].

### Comparative expression profiling of CD24^+^CD90^+^ TICs and non-CD24^+^CD90^+^ control tumor cells

**Figure 3:**
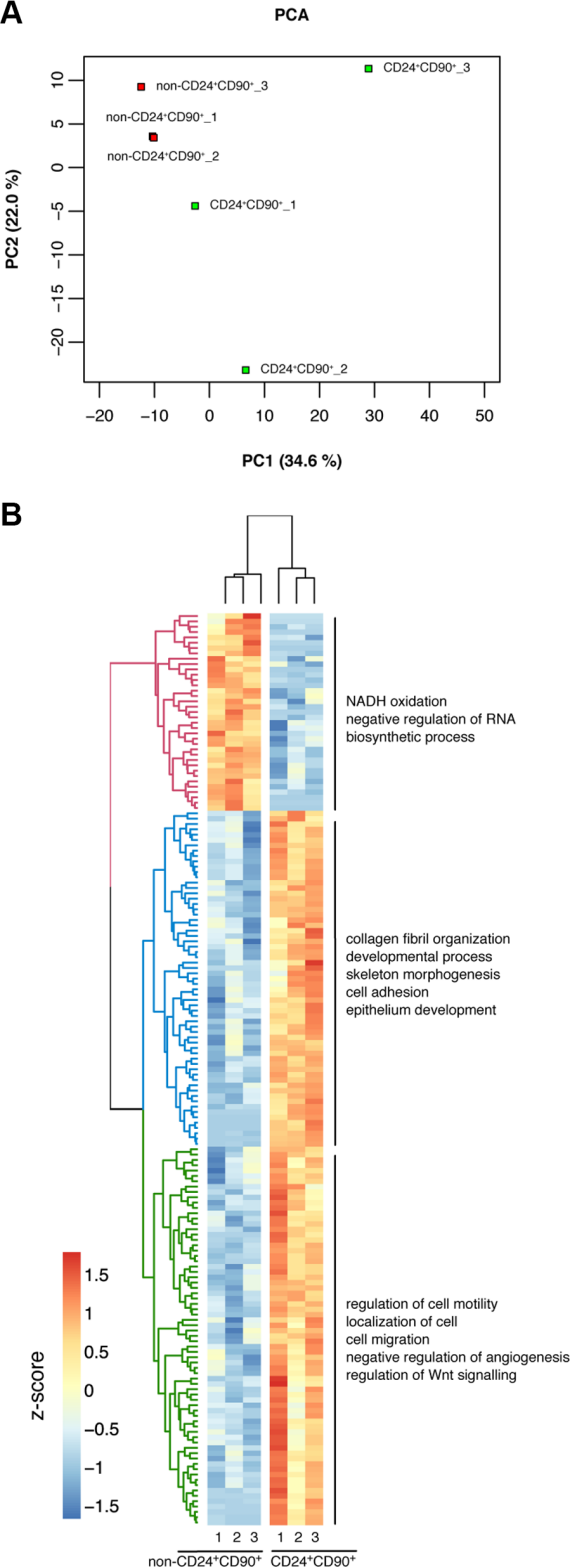
Differential gene regulation in TICs vs non-CD24^+^CD90^+^ tumor cells (**A**) Principle component analysis (PCA) of TICs and non-CD24^+^CD90^+^ tumor cells in triplicates; (**B**) Heatmap of 188 differentially expressed genes in TICs and non-CD24^+^CD90^+^ tumor cells in triplicates. Genes were hierarchically clustered using complete linkage by their Euclidean distance of the scaled and mean centered expression across all samples (z-score). The three main clusters, denoted by color, are additionally annotated by the most representative GO terms based on a hypergeometric test.

RNA-seq analysis was performed to investigate expression differences between TICs and control tumor cells at the transcriptome level. RNA was isolated from freshly sorted TICs and non-CD24^+^CD90^+^ tumor cells. Samples with an RNA Integrity Number (RIN) higher than 6 were subjected to RNA-seq in biological triplicates (Supplementary Figure S1A and S1B), each using a pool of tumors from 4 individual mice. A Principle component analysis (PCA) of the transcriptome data revealed a separation of TICs and non-CD24^+^CD90^+^ tumor cells along the first principle component (34.6%), indicating that these are indeed two distinct cell populations with different gene regulation (Figure 3A). While all three replicates of non-CD24^+^CD90^+^ tumor cells clustered together along the second principle component (22.0%), TIC replicates were more spread, indicating a more heterogeneous population. In detail, the analysis detected 12191 expressed genes, 188 of which were detected as significantly differentially expressed between TICs and non-CD24^+^CD90^+^ tumor cells (a detailed list is given in Supplementary Table S1). The heatmap in Figure 3B depicts the scaled mean expression of the 188 differentially expressed genes with a *q*-value < 0.1, yielding a clear clustering between the three replicates of TICs and non-CD24^+^CD90^+^ tumor cells (Figure 3B). A hypergeometric test using Gene Ontology (GO) Terms shows a general gene upregulation in the categories “developmental process”, “epithelial development”, “cell migration” or “regulation of Wnt signaling” in TICs compared to non-CD24^+^CD90^+^ tumor cells. TICs and the far less tumorigenic non-CD24^+^CD90^+^ tumor cells of MMTV-PyMT cancers can be distinguished based on their transcriptome profiles and already revealed a specific gene regulation based on their tumorigenic behavior.

### An EMT and proteolysis signature is significantly upregulated in TICs

**Figure 4:**
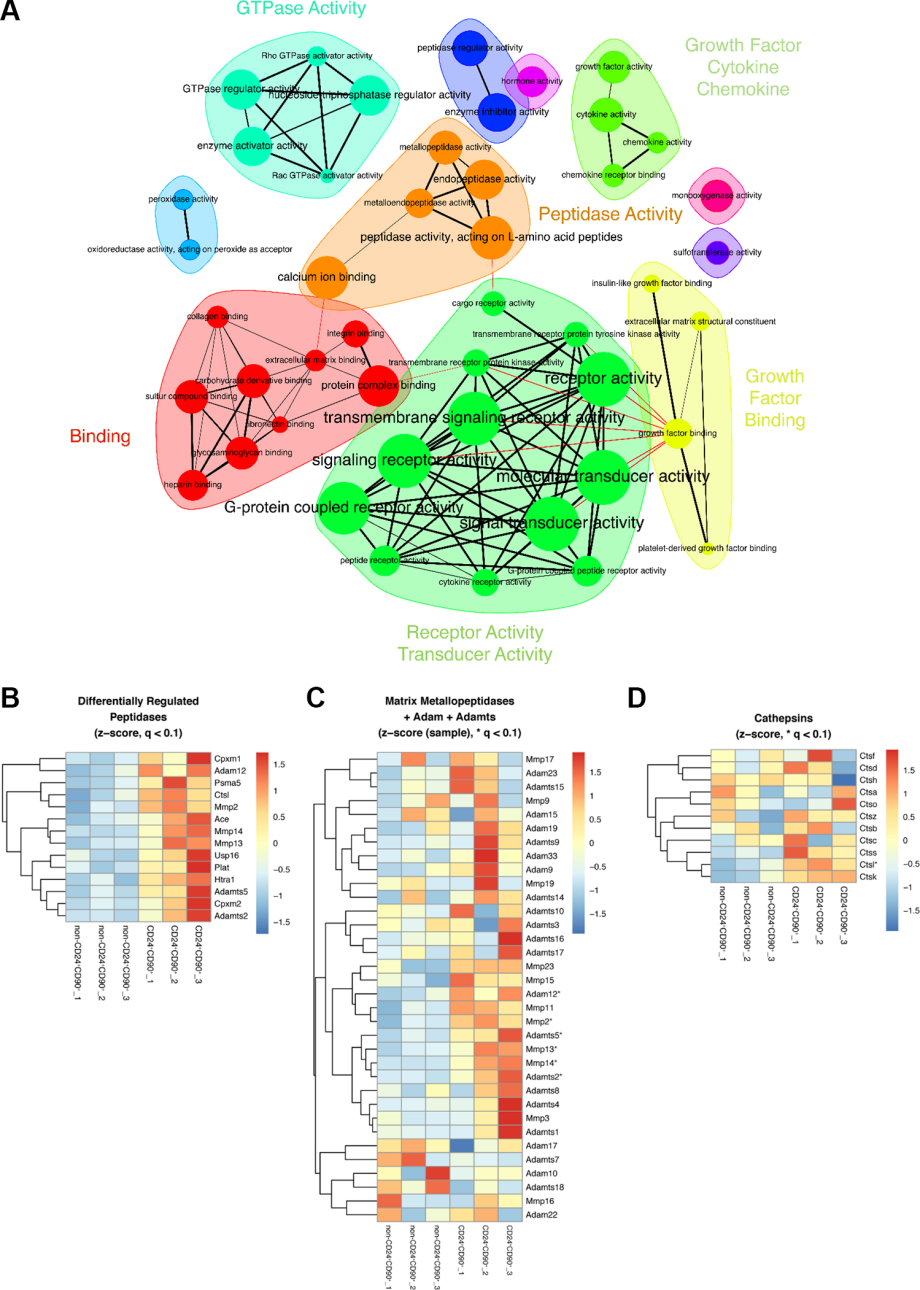
Upregulated gene sets and peptidases in TICs vs non-CD24^+^CD90^+^ tumor cells (**A**) Gene set enrichment analysis (GSEA) of TICs and non-CD24^+^CD90^+^ tumor cells showing upregulated molecular functions in TICs as a network representation. Individual GO Terms are depicted as nodes, which are connected if they share at least 20% of their genes. Node sizes correspond to gene set size; (**B**) Heatmap of significantly upregulated peptidases, *q* < 0.1. Heatmap overview of significantly upregulated peptidases, *q* < 0.1; (**C**) Heatmap of matrix-metallopeptidase (MMP) expression and the disintegrin and metallopeptidase family (ADAMs) expression, and (**D**) Heatmap of cathepsin expression. Significantly upregulated MMPs/ADAMs or cathepsins with *q* < 0.1 are indicated by * in C and D. The heatmaps are clustered row-wise using complete linkage of the respective Euclidean distances.

To gain insight into the molecular pathways operating in TICs, we compared the gene expression using Gene Set Enrichment Analysis (GSEA) (Figure 4A, Supplementary Figures S2 and S3) [27]. Although gene expression analysis of TICs usually focuses on classical stemness signatures, we could not detect any typical stem cell factors (e.g. Oct4, Sox2) in our analysis. Using the mouse Gene Ontology (GO) gene sets, however, upregulation of genes characteristic for epithelial-to-mesenchymal transition (EMT) was observed in TICs compared to non-CD24^+^CD90^+^ tumor cells in accordance with the recently published data by del Pozo et al. [19]. Specifically, the EMT transcription factors Zeb1 (log fold change (logfc) 3.024) and Twist1 (logfc 6.386) were highly upregulated. In addition, the mesenchymal Vimentin (logfc 1.192), several collagens, as well as metastatic niche promoting factors like Periostin (logfc 1.149) and Tenascin (logfc 1.693) were also upregulated in TICs (Supplementary Table S1). These data indicate that MMTV-PyMT TICs undergo an EMT process and support the formation of TIC- derived metastases in the lungs after orthotopic fat pad transplantation of these cells as observed in our study. Although TICs that were cultured on the basement membrane extract Cultrex^TM^ for one week all showed strong E-cadherin staining, some cells presented a mesenchymal phenotype, were less connected and showed development of protrusions (Supplementary Figure S4). This indicates an intermediate EMT state as was recently proposed by del Pozo et al for CD24^+^CD90^+^ TICs. [19] Accordingly, GSEA identified an upregulation of biological processes such as “locomotion and chemotaxis”, and cellular components like “extracellular matrix organization” (Supplementary Figures S2A and S3A).

Furthermore, Figure 4A depicts a network representation of GO gene sets belonging to “molecular function” that are significantly enriched in TICs compared to non-CD24^+^CD90^+^ tumor cells (*q*-value < 0.05). Among the enriched gene sets are “GTPase activity”, “Growth factor binding” and “Receptor/Transducer activity”, which corroborate the colony-forming potential of the TIC population. Strikingly, there was a clear signature for upregulation of protease/peptidase genes in TICs (Figure 4A, shown in orange). More detailed analysis revealed a significant differential upregulation of several proteases, including matrix-metalloproteinases (MMPs), a-disintegrin-and-metalloproteinases (ADAMs), carboxypeptidases, serine and cysteine proteases, a proteasome subunit, and deubiquitinating enzymes (Figure 4B). In contrast, there was no upregulation of any protease in non-CD24^+^CD90^+^ tumor cells. Consistent with these results, MMPs and ADAMs are known to be extensively involved in cleaving the extracellular matrix, thereby contributing to cancer invasion and metastasis [28, 29]. Several MMPs and ADAMs were significantly upregulated including Mmp2, Mmp13 and Mmp14, as well as Adam12, Adamts2, and Adamts5 in TICs (Figure 4C). Cathepsins are another group of proteases involved in tumor progression [30] and, interestingly, Cathepsin L (Ctsl) was significantly upregulated in TICs (Figure 4D).

### MMTV-PyMT TICs possess high extra- and intracellular proteolytic activity and highly express transmembrane Mmp14 on their cell surface

**Figure 5:**
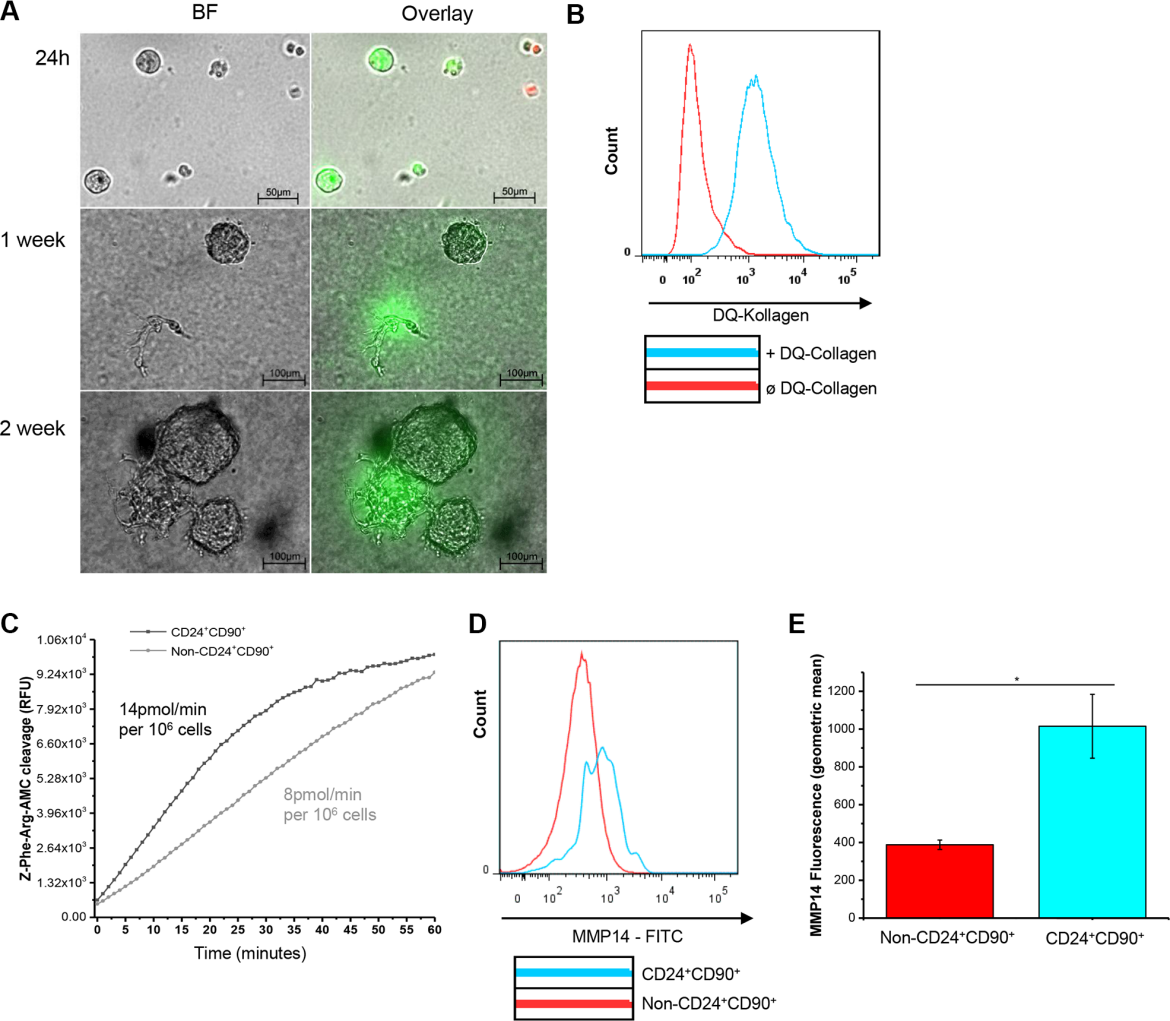
Proteolytic activity of TICs (**A**) DQ-collagen IV^TM^ proteolysis by TICs grown in Clutrex^TM^ for 24 h, one week and two weeks, resp., BF: bright field, green fluorescence: DQ collagen cleavage intracellularly and extracellularly, red fluorescence: PI staining; (**B**) FACS analysis of DQ-collagen IV^TM^ uptake by TICs grown in Clutrex^TM^ for 5 weeks; (**C**) Proteolytic activity of cysteine cathepsins in TICs and non-CD24^+^CD90^+^ tumor cells (both lysates) measured by the conversion of Z-Phe-Arg-AMC, in RFU, velocity was measured over the first 20 minutes; (**D**) Cell surface analysis of transmembrane Mmp14 expression on TICs and non-CD24^+^CD90^+^ tumor cells by flow cytometric analysis; (**E**) Geometric mean of Mmp14 fluorescence of TICs and non-CD24^+^CD90^+^ tumor cells ‘surface. * indicates a significance of *p* < 0.01.

Because of the transcriptional upregulation of proteases in TICs, the proteolytic capacity of those cells was analyzed. Please note that non-CD24^+^CD90^+^ tumor cells showed extremely low frequency of colony formation (Figure 1D). Therefore, non-CD24^+^CD90^+^ tumor cells could not be investigated in the subsequent experiments. Freshly sorted TICs were seeded into 3D cell culture assays consisting of Cultrex^TM^ matrix supplemented with 4% DQ-collagen IV^TM^ [31]. TIC- derived proteases cleaved the DQ-collagen IV^TM^ intracellularly so that a previously quenched fluorescence signal could be detected after 24 h of colony formation (Figure 5A). The TIC colonies increased in size for up to two weeks in culture. Thereafter, the colonies started to form mesenchymal protrusions characterized by extensive extracellular proteolytic activity, again indicating a shift towards a more mesenchymal phenotype (Figure 5A, bottom panel). FACS analysis after five weeks showed increased uptake and intracellular degradation of DQ-collagen IV^TM^ by TICs, indicating proteolysis by endosomal/lysosomal proteases such as cysteine cathepsins (Figure 5B) [32, 33]. To address this further, we performed a biochemical measurement of general cysteine cathepsin enzyme activity in TICs and non-CD24^+^CD90^+^ tumor cells. Strikingly, TICs had a higher substrate turnover (14 pmol/min per 10^6^ cells) compared to the less tumorigenic cells (8 pmol/min per 10^6^ cells) (Figure 5C). To validate the high gene expression of transmembrane protease Mmp14 in TICs compared to non-CD24^+^CD90^+^ tumor cells, as observed in the gene expression analysis (Figure 4C and 4D), its abundance at the cell surface was analyzed by analytical flow cytometry. In line with the mRNA studies this analysis revealed a higher geometric mean value, thus a higher abundance, for Mmp14 at the surface of TICs compared to non-CD24^+^CD90^+^ tumor cells (Figure 5D and 5E). In conclusion, high extra- and intracellular proteolytic activity as well as high expression of transmembrane Mmp14 are hallmarks of CD24^+^CD90^+^ TICs in the MMTV-PyMT breast cancer model.

### Protease inhibitors influence TIC behavior and proteolytic activity

**Figure 6:**
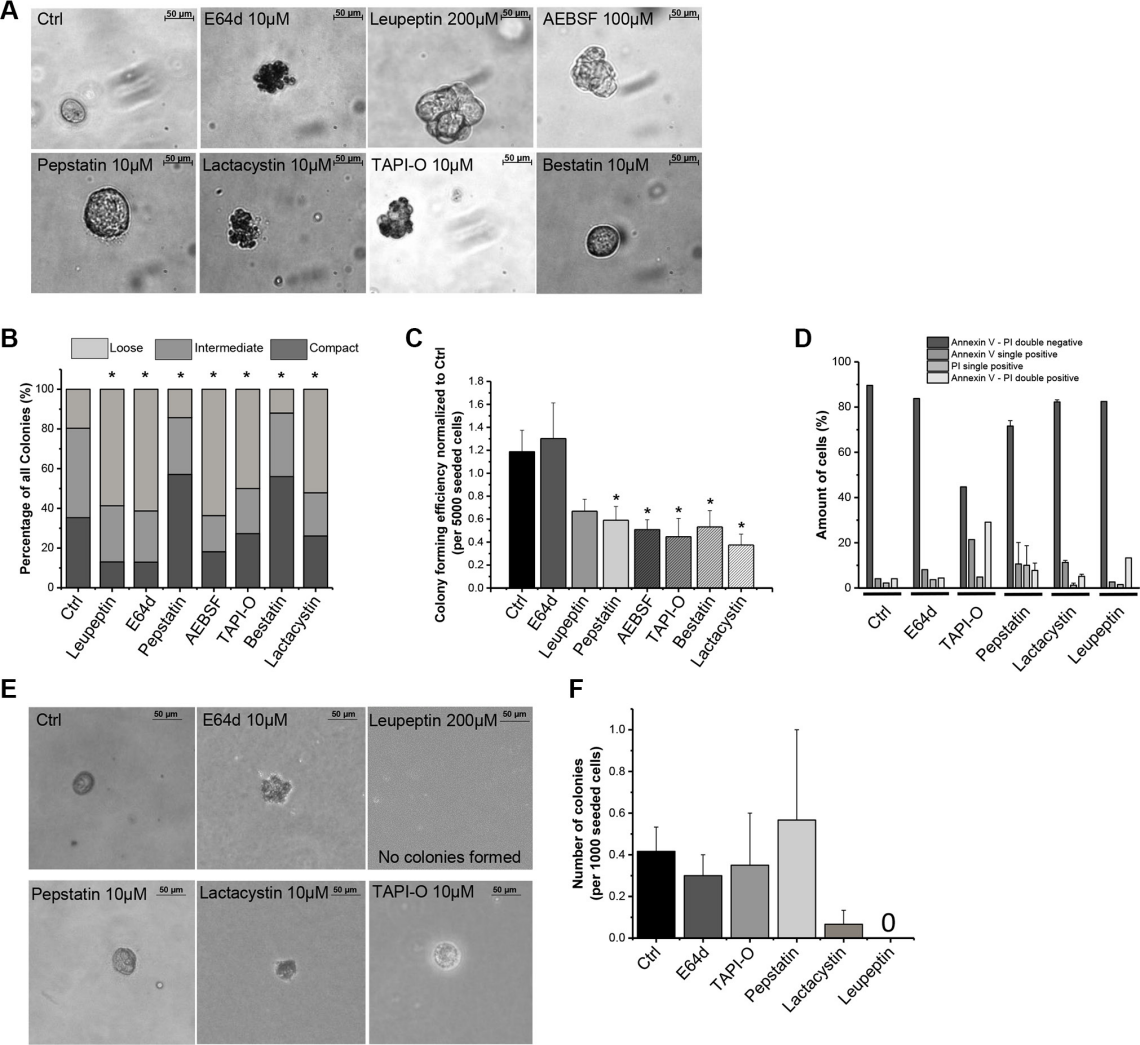
Influence of protease inhibitors on TIC properties (**A**) Morphological changes of TIC-derived colonies upon treatment with broad range protease inhibitors in soft agar (*n* = 3–6); (**B**) Influence of broad range protease inhibitors on morphology of TIC-derived colonies grouped into loose, intermediate and compact colonies; (**C**) Influence of a broad range of protease inhibitors on colony forming efficiency in soft agar of TIC-derived colonies (*n* = 3–6); (**D**) Annexin V – PI analysis of TICs after colony formation assay with broad range protease inhibitors; (**E**) TIC-derived colonies after 4 weeks of secondary colony formation assay; (**F**) Numbers of TIC-derived colonies after 4 weeks of secondary colony formation assay per 1000 seeded cells. Protease inhibitors: E64d, cysteine Cathepsins; Leupeptin, serine, cysteine proteases; Pepstatin, aspartic proteases; AEBSF, serine proteases; TAPI-O, MMPs; Bestatin, aminopeptidases, leukotriene and hydrolases; Lactacystin, proteasome.

To investigate whether protease activity plays a functional role in TICs, several broad range protease inhibitors were applied and anchorage independent growth of TICs was monitored. Some protease inhibitors influenced the morphology of the colonies. While the aspartic protease inhibitors Pepstatin and Bestatin did not affect colony morphology, the cysteine protease inhibitors E64d and Leupeptin, the serine protease inhibitor AEBSF, the proteasome inhibitor Lactacystin and the metalloprotease inhibitor TAPI-O all forced TICs to form loosely connected aggregates instead of compact colonies (Figure 6A and 6B). While control treatment resulted in only 19.6% loose colonies, the amount of loose colonies was shifted to 58.7% by E64d, 61.3% by Leupeptin, 63.6% by AEBSF, 50% by TAPI-O, and 52.2% by Lactacystin treatment (Figure 6B). Colony forming efficiency was greatly reduced by all protease inhibitors except E64d. Lactacystin, Bestatin, and TAPI-O had a great influence on the reduction in colony numbers (Figure 6C), indicating a role in colony formation and tumor growth functions for the proteasome, aminopeptidases, and metalloproteases. Importantly, subsequent analysis of cell death by flow cytometric detection of annexin V binding / propidium iodide (PI) uptake revealed that most of the inhibitors only impaired colony formation but did not result in dead cells. Only TAPI-O treated colonies showed a slight increase in apoptotic and dead cells (Figure 6D). In a second round of colony formation, TICs were able to regrow colonies, although smaller, even in the presence of protease inhibitors E64d, Pepstatin, and TAPI-O. Only Lactacystin and Leupeptin treatment reduced and inhibited, respectively, a second round of colony formation in TICs (Figure 6E and 6F), indicating that the proteasome as well as serine and cysteine proteases are of great importance for the growth abilities of those TIC derived colonies.

**Figure 7:**
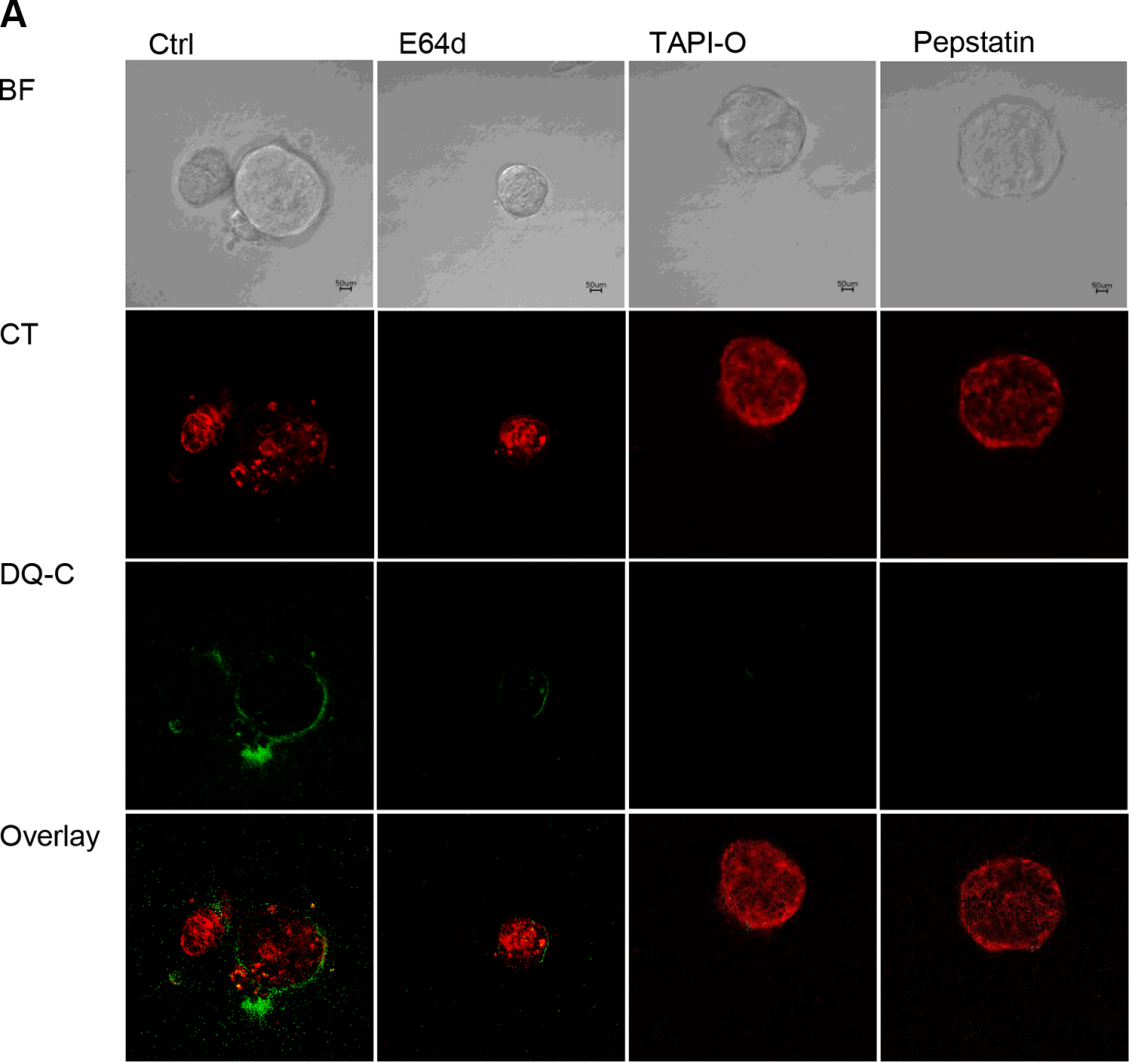
Influence of protease inhibitors on TIC dependent collagen cleavage (**A**) DQ-collagen IV^TM^ assay of TICs in presence of protease inhibitors E64d, TAPI-O and Pepstatin, BF, bright field; red fluorescence, cell tracker red; green fluorescence, DQ-collagen IV^TM^ degradation extracellularly; overlay. Protease inhibitors: E64d, cysteine Cathepsins; Pepstatin, aspartic proteases; TAPI-O, MMPs.

We subsequently tested whether extracellular proteolysis can be reduced upon treatment with protease inhibitors. Therefore, E64d, TAPI-O, and Pepstatin were added to TICs in the 3D DQ-collagen IV^TM^ assay. TAPI-O and Pepstatin greatly reduced cleavage of the quenched collagen resulting in decreased fluorescence signal. E64d did not result in such strong inhibition of DQ-collagen IV^TM^ cleavage (Figure 7A).

These results demonstrate that protease activity is affecting the cancer cell mass initiation of TICs by influencing colony growth and morphology. Further we conclude that the main contributors to extracellular proteolysis of murine breast cancer TICs are metalloproteases, such as Mmp14, Mmp2, and Mmp13 along with aspartic proteases, because treatment with TAPI-O and Pepstatin strongly reduced DQ-collagen cleavage.

## DISCUSSION

In this study we demonstrate that CD24^+^CD90^+^CD45^−^ cancer cells of the MMTV- PyMT breast cancer model possess high tumorigenicity in clonogenic *in vitro* assays as well as *in vivo* upon transfer of only 100 cells into the mammary fat pad of immunodeficient mice. These findings support that CD24^+^CD90^+^CD45^−^ cells are a *bona fide* TIC population. This is in line with previous findings in the MMTV-Wnt 1 breast cancer model [15]. The markers used in this study have been shown to enrich normal mouse mammary stem cells, especially CD24 [34, 35]. CD90 is a hematopoietic stem cell marker that has been shown to label a subset of CD24^+^ cancer stem cells [36, 37]. This resemblance supports these markers to identify TICs also in breast cancer.

The fact that marker sets for TIC populations in cancers differ suggest that there are specific marker combinations for every murine breast cancer model and subtype of human mammary carcinomas. The p53-null mammary tumor model possesses a cell population positive for CD24 and CD29 (Lin^−^CD24^H^CD29^H^) that is tumorigenic and recapitulates the primary tumor [38]. A marker set including CD24^+^CD29^+^CD61^+^ identifies yet another group of TICs in this model. TICs of the MMTV-Wnt1 mouse model with the same marker profile generated tumors upon orthotopic injection of only a few cells [14]. However, it is important to determine the tumor- and metastasis- initiating capabilities of the various marker-defined TIC populations in order to identify molecular patterns enabling the pro-malignant actions of those cells.

Gene expression analysis of TICs focuses primarily on stem cell factors and EMT signatures [4, 15, 39]. When characteristic stem cell signatures, including stem cell markers and self-renewal genes, were searched for in our transcriptome profiles of MMTV-PyMT TICs, they were not detected nor found to be differentially regulated. However, concordant with the recently published data by del Pozo et al. an upregulated EMT signature was found [19]. These data indicate that MMTV-PyMT TICs undergo an EMT process with intermediate stages, which could be due to heterogeneous sub-populations within CD24^+^CD90^+^ cells and has been proposed recently also for human breast cancer TICs [26, 32–34]. Importantly, in our study the CD24^+^CD90^+^ TICs implanted into mammary glands were able to form macrometastases in lungs, therefore supporting the concept of EMT-generated metastatic cancer stem cells / TICs [12, 19, 40].

A functional proteolytic network is known to drive cancer progression at all tumor stages [1, 20, 21]. Strikingly, our study demonstrates that TICs are characterized by a significantly upregulated proteolytic signature compared to the non-CD24^+^CD90^+^ tumor cells of this model. Significantly upregulated proteases include members of the MMP family, ADAM and ADAMTS family, as well as cathepsin L (Ctsl). Proteases have long been known to be involved in extracellular matrix (ECM) degradation, as well as in the liberation of intact cytokines, chemokines, and growth factors from the ECM. Thus, proteases promote proliferation, cell differentiation, and cell detachment, as well as EMT and angiogenesis in physiological and cancer settings [42–45].

High gene expression and protein levels of MMPs are associated with advanced tumor grade, risk of metastasis, and tumor recurrence [46]. In the MMTV-PyMT mouse model Mmp14 is required for efficient tumor dissemination [47]. Furthermore, Mmp2, Mmp9, and Mmp13 become upregulated in MMTV-PyMT tumors and are also involved in tumor growth and metastasis [48, 49]. Supporting these findings, a significant upregulation of Mmp2, 13, and 14 via RNA-seq analysis of MMTV-PyMT TICs was identified in this study. In addition a higher cell surface abundance of Mmp14 on TICs compared to non-CD24^+^CD90^+^ cells was observed. Further, a CD90^+^ pool of cells able to initiate tumor and metastasis in esophageal cancer as well as CD24^+^CD90^+^ mesenchymal initiating cells (MICs) also show an upregulation of Mmp 2, 9, 13 and 14 [19, 50]. Inhibition of those MMPs reduced colony formation as well as collagen degradation *in vitro*. Due to their important role in cancer, attempts to design synthetic and natural inhibitors for MMPs were successfully established in preclinical trials, but not beyond phase II at later stages of the disease [42, 51]. However, one should consider MMPs specifically as a target in the TIC population to reduce tumor promoting and possibly metastatic abilities of these cells, as was shown *in vitro* by TAPI-O inhibition.

A further group of metalloproteinases, the ADAMs and ADAMs with a thrombospondin motif (ADAMTS), are highly expressed at the invasive fronts of tumors and correlate with poor prognosis [52, 53]. Due to their sheddase activity cleaving off transmembrane proteins, including EGF-receptor ligands, Fibroblast growth factor receptor, CD44, E-cadherin, N-cadherin, Notch, L1, and TNF-α, they regulate paracrine and autocrine signaling pathways involved in tumor growth and progression [52, 54–56]. ADAMTS1, as well as ADAMTS8 and 15, all promote tumor growth and metastasis in the MMTV-PyMT model, and predict survival of human basal breast cancer [57, 58]. We complement those findings by the detection of Adam12, Adamts2 and Adamts5 as being significantly upregulated in MMTV-PyMT TICs. Due to increasing evidence for tumor-promoting functions of various ADAMs and ADAMTS in multiple cancer entities, therapeutically targeting this group of proteases has to come into focus [52].

Like the other two groups of proteases, lysosomal cysteine cathepsins (Cts) correlate with poor clinical outcomes in several cancers [59, 60]. Instead of acting in the lysosome as their normal cellular compartment, they can be secreted to function in distinct extracellular roles in the tumor microenvironment [21, 61, 62]. Upregulated or highly active Cts in human cancers and tumor mouse models include Cts B, C, D, H, L, S, and X/Z [63–65]. In the MMTV-PyMT mouse model overexpression of human Cathepsin B and Cathepsin L promoted migration and invasion of cancer cells as well as metastasis formation [66, 67]. In this study, high expression of Ctsl in TICs has been observed and found to influence TIC behavior and possibly TIC metastasis to the lungs.

The small population of TICs that is obtained from the MMTV-PyMT mouse model (between 3000 and 10000 TICs per mouse) makes it difficult to investigate the upregulated proteases by standard cell biological methods. Nevertheless we were able to show collagen cleavage abilities which can be influenced by protease inhibitors, high cathepsin B/L activity by cleavage of their specific substrate Z-Phe-Arg-AMC, and Mmp14 surface abundance in TICs. The establishment of a TIC line derived from primary isolated TICs could help to uncover the specific roles of the involved proteases in more detail.

In summary, we show that CD24^+^CD90^+^CD45^−^ TICs possess a proteolytic gene signature and high proteolytic activity. When treated with a broad range of protease inhibitors, anchorage independent growth of TICs was reduced and their morphology was influenced. Due to the upregulation of several protease families in TICs it is necessary to closely investigate their individual functions and target not only one specific protease, but rather several proteases that work together in a proteolytic network [20, 68]. Our results suggest proteolysis to be considered as a novel characteristic of TICs in which proteases should not be considered as oncogenes initiating tumor growth but rather promote tumor growth through enabling micro-motility of TICs through the ECM [69].

## MATERIALS AND METHODS

### Animal model

FVB/N-TgN(MMTVPyVT)634Mul/J [70] mice were used as a model for invasive metastasizing breast cancer. Lymphocyte deficient Rag2^−/−^ γc^−/−^ female mice [71, 72] were used for orthotopic transplantations. The maintenance of the animals as well as the orthotopic transplantation experiments were performed in accordance to the German law for animal protection (Tierschutzgesetz) as published on May 18^th^ 2006.

### Tumor harvest and single cell suspension

Tumors were harvested from 12 or 14 week old MMTV-PyMT female mice. After mechanical disruption, tumor pieces were enzymatically digested with 0.25 mg/ml DNAse I, 6 mg/ml Collagenase IV, and 1 mg/ml Hyaluronidase I (all Sigma, St. Louis, MO, USA) for 1 h at 37°C. After digestion, samples were filtered twice with 100/70 μm cell strainers (BD, Franklin Lakes, NJ, USA). Afterwards, erythrocytes were lysed (0.15 M NH4Cl, 1 mM KHCO3 and 0.1 M EDTA). Cells were resuspended in PBS, counted and used for either FACS sorting or cell culture experiments.

### Cell staining and FACS sorting/analysis

Cells were resuspended in FACS buffer (PBS, 2% FCS and 5 mM EDTA) and stained at a concentration of 1 × 10^4^ cells/μl. After blocking of unspecific antibody binding with CD16/32 (BD Pharmingen, Franklin Lakes, NJ, USA), specific antibodies were added at appropriate dilutions (CD24PE, clone M1/69, BD Pharmingen; CD90PE-Cy5, clone HIS51, eBioscience, San Diego, CA; CD45PE-Cy7, clone 30-F11, eBioscience) for 30 min on ice in the dark, followed by addition of 4′,6-Diamidin-2-phenylindol (DAPI). Stained cells were then washed and resuspended in 200-1000 μl FACS buffer. For Mmp14 staining, cells were first incubated with the primary antibody, anti-Mmp14, clone EP1264Y, and subsequently with the secondary antibody goat-anti rabbit FITC, both Abcam, Cambridge, UK, at appropriate dilutions.

Sorting and analysis of cells was performed using a FACSAriaIII or Fortessa flow-cytometer, with Diva (BD Bioscience, Franklin Lakes, NJ, USA) and FlowJo (FlowJo, LLC, Ashland, OR, USA) software. Forward and side scatter profiles, depletion of DAPI positive, as well as CD45 positive cells were used as selection criteria. Cells that were either positive for both CD24 and CD90 or negative for either or both markers were analyzed for Mmp14 cell surface expression, and collected.

### Orthotopic transplantation

Either sorted CD24^+^CD90^+^ or non-CD24^+^CD90^+^ tumor cells at dilutions of 100, 1000 or 4000 cells were resuspended in 25 μl PBS and mixed with an equal amount of Cultrex^TM^ (Trevigen, Gaithersburg, MD, USA). The cell/Cultrex^TM^ mixture was then transferred into the fat pad of the fourth mammary gland via a 5 mm lateral incision. Tumor growth was monitored by palpation twice a week for 5 months in total, followed by tumor harvest when about 1 cm^3^, and further analysis.

### Histology and Immunohistology

Isolated tumors and lungs were fixed in 4% paraformaldehyde and paraffin-embedded. 5 μm sections were obtained, stained with Hematoxylin/Eosin (both Sigma), as well as Ecadherin (BD Pharmingen), and Ki67 (Santa Cruz, Heidelberg, Germany). Detection of primary antibody was performed using the Vectastain Elite ABC kit (Vector Laboratories, Burlingham, USA), followed by 3,3-diaminobenzidine (DAB) incubation. Slides were analyzed by light microscopy (Zeiss, Oberkochen, Germany).

### Colony formation assay

Up to 5000 sorted TICs, non-CD24^+^CD90^+^ tumor cells or whole tumor cells subjected to live/death sorting, resuspended in a top agar of 0.7% noble agar (Sigma) and DMEM supplemented with 20% fetal calf serum (FCS) (PAN, Aidenbach, Germany), 2% penicillin/streptomycin (P/S) and 4 mM L-glutamine (both Gibco/Invitrogen, Waltham, MA, USA), were seeded onto a base agar of 1% per well. After solidification of the agar, DMEM containing 10% FCS, 1% P/S and 2 mM L-glutamine was added. Cells were either treated with culture medium with or without either DMSO or protease inhibitors E64d (10 μM, Bachem, Weil am Rhein, Germany), Leupeptin (200 μM, Fluka- Sigma), 4-(2-Aminoethyl) benzensulfonylfluorid (AEBSF) (100 μM, Sigma), Pepstatin (10 μM, Sigma), Lactacystin (10 μM, EnzoLifeScience, Lausen, Switzerland), TAPI-O (10 μM, EnzoLifeScience) or Bestatin (10 μM, Gbioscience, St Lousi, MO, USA) every second day. The culture was kept at 37°C for 4 weeks. A 2^nd^ CFA was performed, after isolating the colonies from agar and preparing a single cell suspension, the same way. Cell numbers seeded in a 2^nd^ CFA varied depending on the amount that was obtained from the first CFA.

### Annexin V – propidium iodide flow cytometric analysis

Colonies from the CFA were isolated from soft agar via dilution in warm PBS and centrifugation. Single cells were obtained via incubation with Accutase, Merck Millipore, Billerica, MA, USA, for 15 minutes at 37°C, subsequently washed in PBS and resuspended in 1x- Annexin V binding buffer. Cells were incubated for 15 minutes with Annexin V-FITC, Santa Cruz Biotechnology, Dallas, TX, USA, (0.1–1μg). Shortly before FACS analysis, propidium iodide was added at appropriate dilutions and samples were analyzed on a FACS Calibur, BD.

### Immunofluorescence

Freshly sorted TICs we seeded onto a thin layer of Cultrex^™^ (Trevigen) / DMEM containing 10% FCS, 1% P/S and 2 mM L-glutamine, in a 1:1 mix, and cultured for one week at 37°C. TICs were then fixed with 4% formaldehyde for 30 min, made permeable with 0.2% Triton X-100 for 7 min at room temperature (RT), and fixed with ice cold Acetone for 4 min at −20°C. Subsequently, TICs were blocked by BSA for 30 min at RT and the primary E-cadherin antibody (BD Bioscience, clone 36) was applied at appropriate dilutions overnight at 4°C. After washing, the secondary antibody anti-mouse Alexa fluor 546 (Life technologies) was applied for 1 h at RT. Nuclei of cells were stained with Hoechst dye for 5 min at RT.

### Transcriptome analysis by RNA-seq

RNA was isolated from TICs or non-CD24^+^CD90^+^ tumor cells using the Absolutely RNA Nanoprep Kit (Agilent, Basel, Switzerland). Its quality was assessed on an Agilent Bioanalyzer 2100 (Agilent). Samples (120 ng RNA) were subject to RNA-seq in triplicate, each using a pool from 4 cell sorts for either TICs or non-CD24^+^CD90^+^ tumor cells. RNA-seq was done by Eurofins MWG operon service by sequencing libraries on an Illumina HiSeq 2000 instrument (Eurofins, Munich, Germany). Raw sequence reads were first subjected to trimming using Trimmomatic [73] (version 0.32), where adapter sequences and low quality bases were removed. Afterwards, reads were aligned to the GRCm38 mouse genome using the STAR [74] alignment algorithm (version 2.4.0). Alignment post-processing included the creation of bam-files, sorting of reads and the removal of non-uniquely mapped reads from further analysis and was carried out using samtools [75] (version 1.1). Read-counts per gene were calculated with HTSeq [76] and subsequently loaded into the statistical computation environment R [77]. For the analysis of the count-data we used the edgeR package [78] as proposed by Anders et al. [79]. First, weak intensity features were removed by thresholding the counts per million reads (cpm) value. Differential gene expression was assessed using the *exactTest* method of edgeR and resulting *p*-values were corrected for multiple testing using the method of Benjamini and Hochberg [80]. The sequence data has been deposited at Gene Expression Omnibus (GEO) under the ID GSE75946.

### Enzyme activity assay

Cells were lysed in 100 mM sodium acetate, 1 mM EDTA, 0.05% Brij, and 1 mM DTT and mechanically disrupted using a 22 gauge-needle (Braun, Melsungen, Germany). The fluoropeptid Z-Phe-Arg-7-Amino-4-methylcoumarin (AMC) (Bachem) was added to the samples of 37°C (0.5 nM). Its release was measured every minute for 60 minutes at excitation and emission wavelengths of 360 and 460 nm, respectively. Enzyme activity was normalized to cell number.

### 3D cell culture and DQ collagen assay

TICs or CD24^+^CD90^+^ normal mammary gland cells were suspended in culture medium (150 μl/well), mixed with an equal volume of Cultrex^TM^ and seeded onto low adhesion 24 well plate or 8 well Ibidi plate (Ibidi, Martinsried, Germany). For imaging of proteolytic activity, DQ-collagen IV^TM^ (LifeTechnologies, Carlsbad, CA, USA; λ_em_ = 488 nm) was added and the well coated prior to seeding. After solidification (1 h at 37°C), culture medium was added on top. Depending on the experiment, culture medium contained protease inhibitors as previously mentioned. Cultures were kept under hypoxic conditions (3% O2, 5% CO_2_, 92% N_2_, 37°C).

### Data presentation and statistical analysis

Quantitative data are presented as mean ± S.E.M. Statistics was performed by *t-test* for two group-comparisons, Log-Rank test for testing for equality of groups or Chi Square test (software OriginPro 8.6; OriginLab Corporation, Northampton, MA, USA). Limiting dilution frequencies were calculated with L-Calc^TM^ software (Stemcell Technologies, Vancouver, Canada).

## SUPPLEMENTARY MATERIALS FIGURES




