# Supplementary material for: Proteolysis-a characteristic of tumor-initiating cells in murine metastatic breast cancer
Source: Oncotarget. 2016 Aug 16;7(36):58244–60. doi: 10.18632/oncotarget.11309 (PMC5295428; doi:10.18632/oncotarget.11309)
Supplement: Supplementary file 1 [file oncotarget-07-58244-s001.pdf]

Proteolysis – a characteristic of tumor-initiating cells in murine metastatic breast cancer

Supplementary Materials

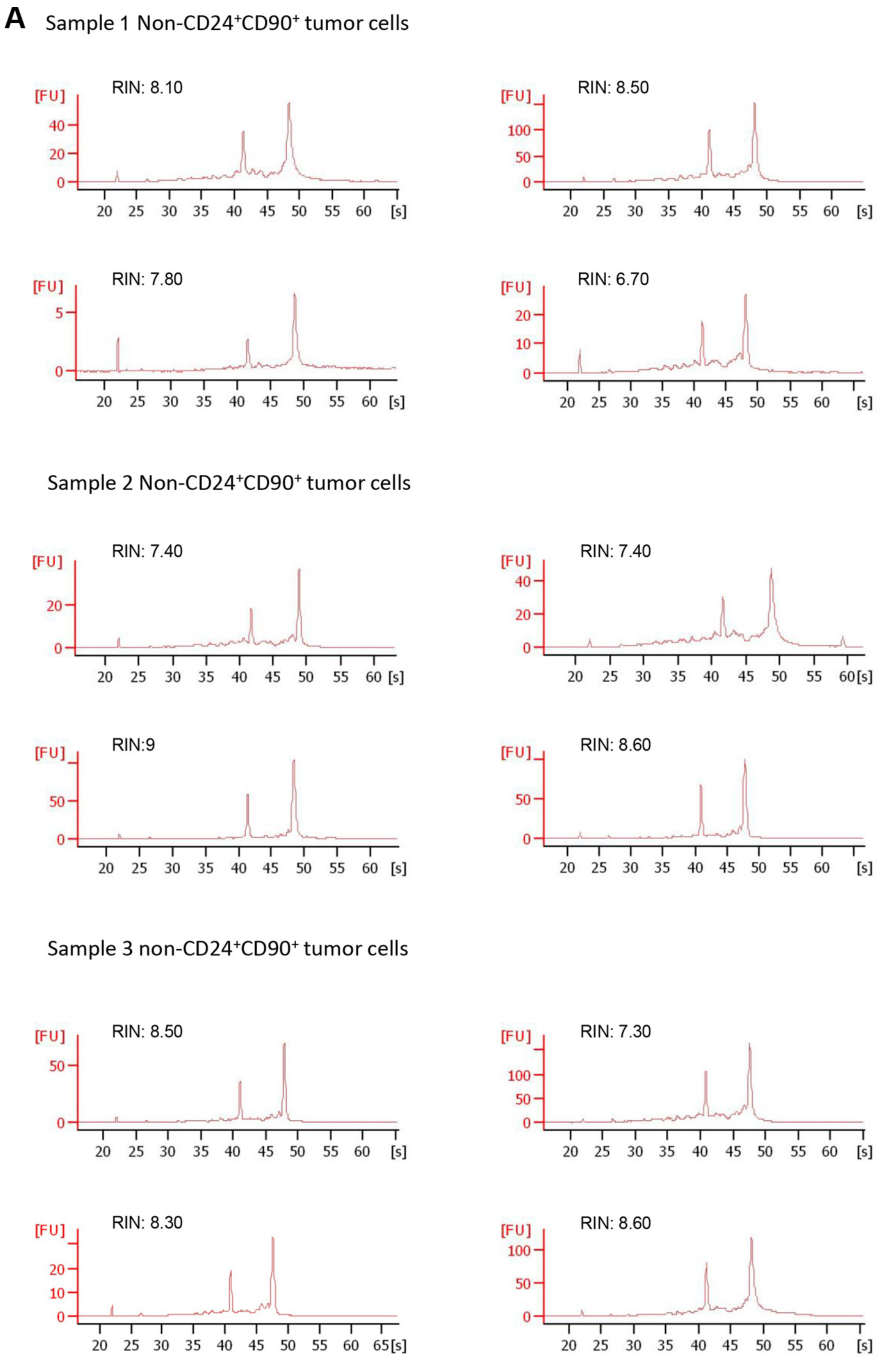

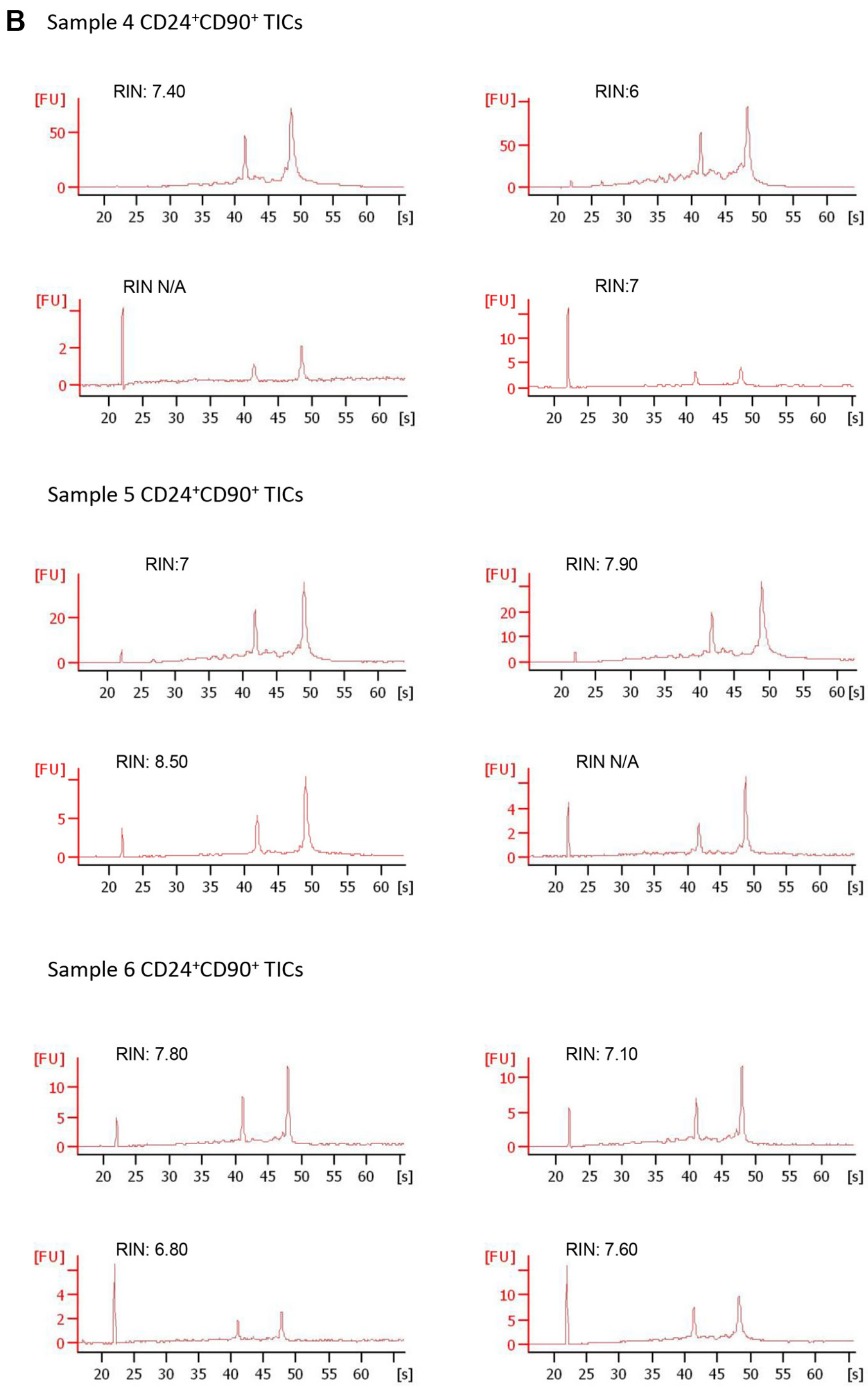

Supplementary Figure S1: RNA quality control of TICs and non-CD24^{+}CD90^{+} tumor cell samples. (**A**) Analysis of RNA Integrity Number (RIN) for RNA quality control of non-CD24^{+}CD90^{+} tumor cells; and (**B**) TICs from four different sortings per triplicate sample, resulting in a total of 12 mice. A RIN of 6 was considered for inclusion in RNA-seq analysis.

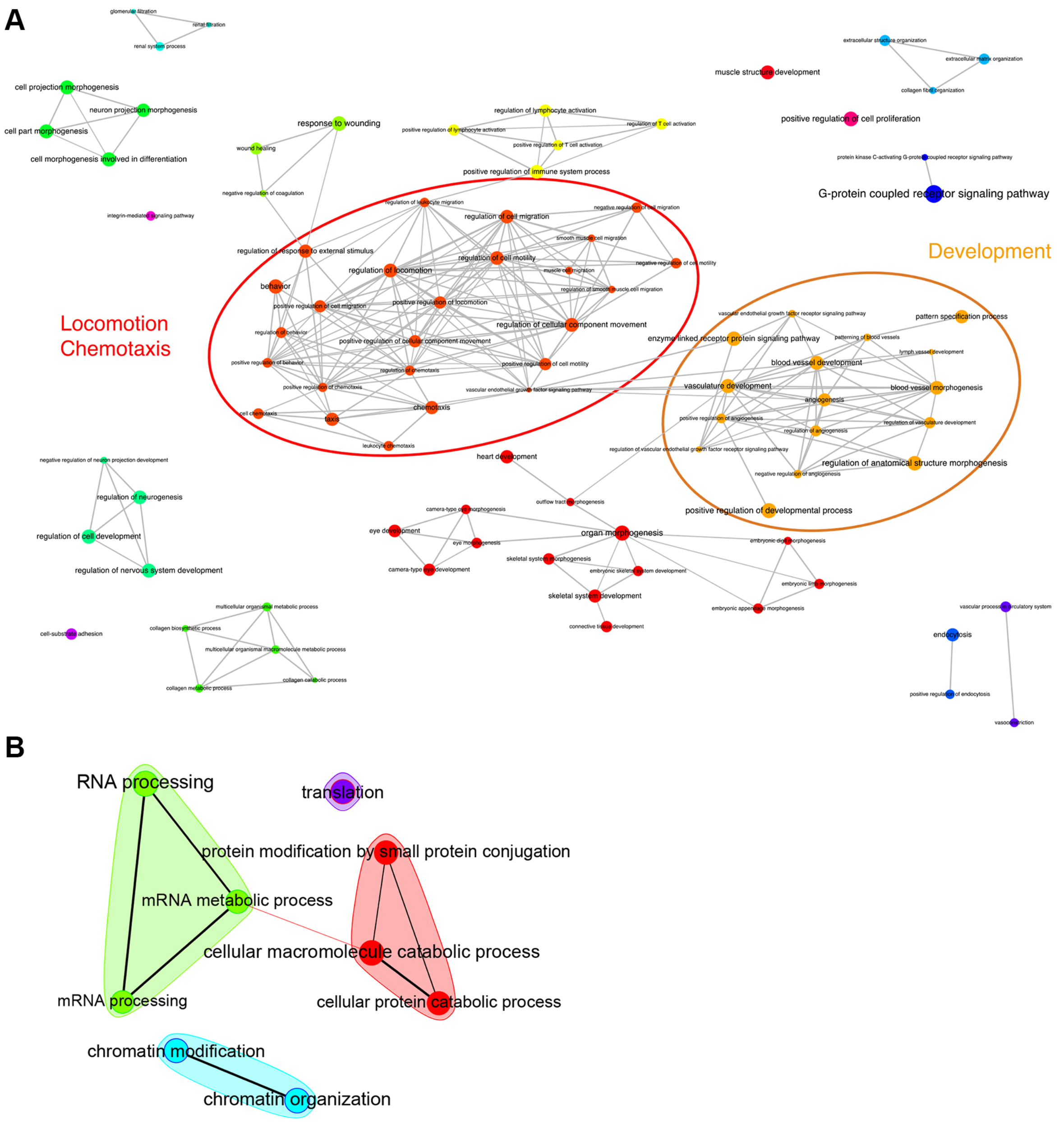

Supplementary Figure S2: Over- and underrepresented gene sets in TICs vs non-CD24^{+}CD90^{+} tumor cells. Gene set enrichment analysis of RNA-seq gene expression data was performed using GSEA. Displayed are gene sets, which obtained a q-value < 0.05. Overlapping gene sets were connected by an edge if they share at least 70% of their genes. Related gene sets were grouped in colored areas. Shown are biological processes up- (**A**); and downregulated (**B**) in TICs.

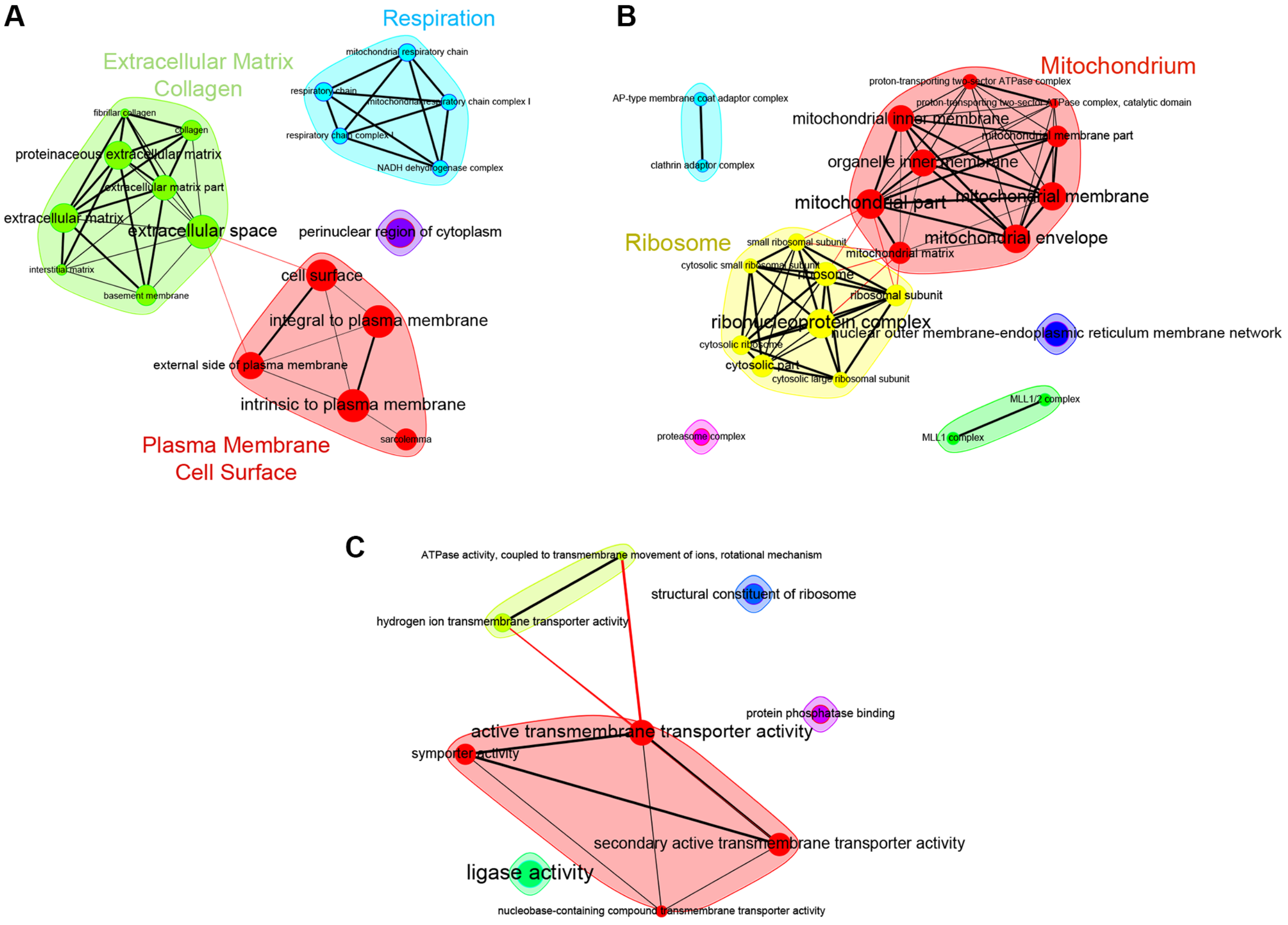

Supplementary Figure S3: Over- and underrepresented gene sets in TICs vs non-CD24⁺CD90⁺ tumor cells. Gene set enrichment analysis of the RNA-seq gene expression data was performed using GSEA. Displayed are gene sets, which obtained a *q*-value < 0.05. Overlapping gene sets were connected by an edge if they share at least 70% of their genes. Related gene sets were grouped in colored areas. Shown are up- (**A**); and downregulated (**B**) cellular components of TICs; as well as downregulated molecular processes in TICs (**C**).

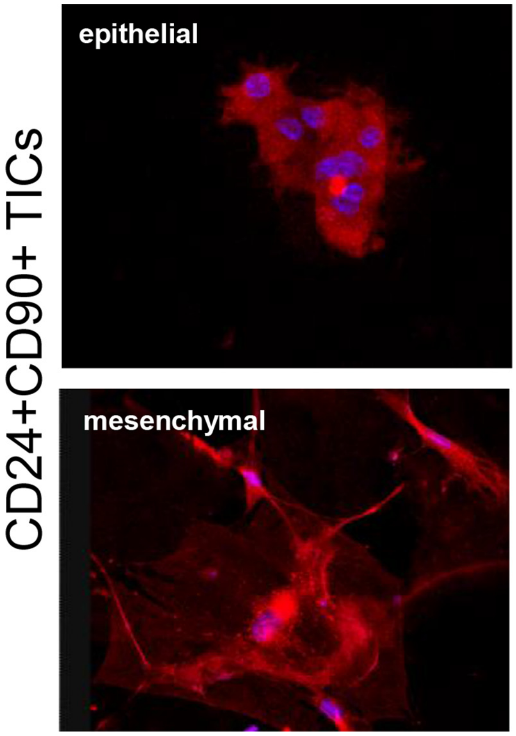

Supplementary Figure S4: CD24⁺CD90⁺ TICs show intermediate stages of EMT. TICs cultured for one week on Cultrex™ show strong E-cadherin staining (red; nuclei blue). However, cells show two types of morphologies: One group of cells is more epithelial and closely connected, while another group of cells developed protrusions and are less connected, indicating a more mesenchymal phenotype.

Supplementary Table S1: List of differentially expressed genes between TICs and non-CD24⁺CD90⁺ tumor cells. 188 genes significantly differentially expressed in TICs compared to non-CD24+CD90+ tumor cells with a *p*- value < 0.01. See Supplementary_Table_S1
